# A new aberrantly spliced *BCR‐ABL1* transcript variant (e13a1) identified in routine monitoring using different quantitative reverse transcription polymerase chain reaction techniques in a patient with chronic myeloid leukemia

**DOI:** 10.1002/jha2.553

**Published:** 2022-09-02

**Authors:** Nicole Naumann, Ulrike Bross‐Bach, Wolfgang Seifarth, Alice Fabarius, Wolf‐Karsten Hofmann, Susanne Saußele, Birgit Spiess

**Affiliations:** ^1^ Scientific Laboratory Department of Hematology and Oncology University Hospital Mannheim Heidelberg University Mannheim Germany; ^2^ Department of Internal Medicine, Clinic of Hematology, Oncology, Clinical Immunology and Rheumatology University Hospital Tübingen Tübingen Germany

**Keywords:** BCR‐ABL1, CML, qRT‐PCR, rare transcript, splice variant

## Abstract

Quantitative reverse transcription polymerase chain reaction (qRT‐PCR) of *BCR‐ABL1* transcript level is an essential part of routine disease monitoring in patients with chronic myeloid leukemia. One patient sample (e13a2 transcript detected by nested PCR) attracted attention by revealing an aberrantly spliced *BCR‐ABL1* transcript variant e13a1. The last 38 base pairs (bp) of *BCR* exon 13 were replaced by a 37 bp insertion of the *ABL1* intron 1–2/exon 1 sequence. The rare aberrant *BCR‐ABL1* fusion transcript can cause discrepancies in molecular diagnostics. This scenario highlights the importance of an individual characterization of the *BCR‐ABL1* fusion sequence in case of unclear qRT‐PCR results.

## 1 INTRODUCTION

The reciprocal translocation between chromosomes 9 and 22 (t(9;22)(q34;q11)) resulting in the Philadelphia chromosome (Ph) and the *BCR‐ABL1* fusion gene is causal to the development of chronic myeloid leukemia (CML). The occurrence of different *BCR‐ABL1* mRNA fusion variants (most commonly e13‐a2, e14‐a2, and e1‐a2) results in the expression of an abnormal BCR‐ABL1 fusion tyrosine kinase in the majority of the patients. In most cases, the breakpoints occur within the major breakpoint cluster region (M‐bcr) within the *BCR* gene. The breakpoints are less often located in two other breakpoint cluster regions, termed minor (m‐bcr) and micro (μ‐bcr). Rare atypical BCR breakpoints outside these cluster regions, novel *BCR‐ABL1* transcripts with insertion and/or deletion of different *BCR* and *ABL* sequence sections, and atypical splicing events were detected [1, 2, 3, 4, 5, 6, 7, 8, 9, 10, 11, 12]. Here, we report on the occurrence of a novel *BCR‐ABL1* transcript generating most likely a functional BCR‐ABL1 tyrosine kinase in a Ph‐positive CML patient where standard diagnostic quantitative reverse transcription polymerase chain reaction (qRT‐PCR) procedure showed no amplification of the typical *BCR‐ABL1* transcripts.

## 2 MATERIALS AND METHODS

A 69‐year‐old female patient was diagnosed with CML in 2005. At diagnosis, the translocation t(9;22)(q34;q11) and the transcript type e13a2 were determined extramurally. The patient samples were obtained with written informed consent in accordance with the declaration of Helsinki, and the analysis was approved by the institutional review board of the Medical Faculty of Mannheim, Heidelberg University (Heidelberg, Germany). The first monitoring sample was investigated in our laboratory 6 months after the start of imatinib therapy. At this time, a qualitative multiplex PCR assay [13] for detection of the regular *BCR‐ABL1* transcript types (e13a2 and e14a2) was also performed in our laboratory in Mannheim. The molecular monitoring of the *BCR‐ABL1* fusion transcript was performed using two standard qRT‐PCR methods (LightCycler [LC] and TaqMan [TM]) for molecular monitoring of usual fusion transcripts [14, 15]. Both methods differ in PCR amplicon length (LC: 596 bp; TM: 228 bp) and primer/probe combinations. In addition, the samples were retrospectively investigated with our nested PCR assay for regular transcripts and our one‐step PCR assay for irregular transcripts. PCR amplicons were characterized by Sanger DNA sequencing.

## 3 RESULTS AND DISCUSSION

For evaluation of the externally transmitted (e13a2) transcript type, we performed our qualitative in‐house multiplex PCR assay as previously described [13].

Since the patient received imatinib therapy over 6 months, the negative multiplex PCR result for any transcript variant could be explainable by a low *BCR‐ABL1* quotient or a binding failure of primer(s) or probe. Therefore, we performed a nested‐PCR assay [16] and we succeeded in the detection of amplicons with typical e13a2 transcript length. To exclude target limiting effects in the nested PCR, we used a one‐step PCR assay for atypical transcripts (in‐house unpublished) with different primer combinations compared to multiplex PCR. These experiments confirmed the positivity of e13a2 transcript length.

**FIGURE 1:**
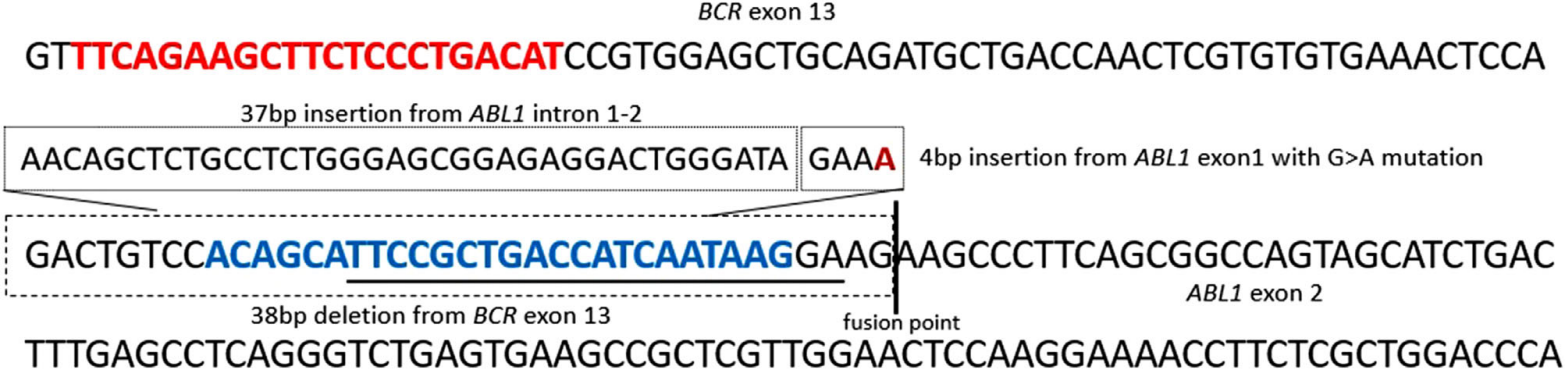
Sequence of the aberrantly spliced *BCR‐ABL1* transcript variant (e13a1). Sense primer binding site for nested and LightCycler PCR systems is shown in red bold letters. The sense primer binding site for multiplex PCR is shown in blue bold letters and the sense primer for the TaqMan PCR system is underlined. Since the primer binding sites for multiplex and TaqMan PCRs are deleted, no PCR amplification products could be generated. However, the primer binding sites for nested and LightCycler PCR systems remained unaffected and, therefore, PCR products were amplified.

**FIGURE 2:**
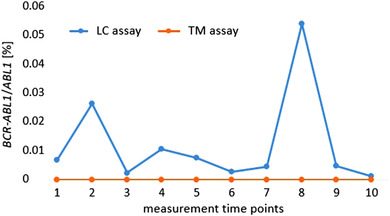
Retrospective measurement of *BCR‐ABL1/ABL1* quotient with in‐house LightCycler (LC) and TaqMan (TM) assays at different time points. Due to the deletion of parts of *BCR* exon 13, the TM forward primer was not able to bind the target sequence resulting in no signal (orange). The LC assay (using another primer/probe combination) can detect the *BCR‐ABL1* fusion transcript (blue).

Sanger DNA sequencing of one‐step PCR products revealed an atypical *BCR‐ABL1* transcript variant (e13a1) as shown in Figure 1. The primer binding site for the BCR sense primer of the TM qRT‐PCR method [15] and the qualitative multiplex PCR assay [13] was deleted (38 bp deletion) and replaced by a 37 bp insertion of *ABL1* intron 1–2/exon 1 sequence. This sequence exchange resulted in missing PCR amplicons. Using the primer/probe combination of the LC qRT‐PCR method [14], PCR amplicons were detectable (Figure 2). The binding site of the BCR sense primer was located 73 base pairs further upstream and was, therefore, not affected by the deletion/insertion.

**FIGURE 3:**
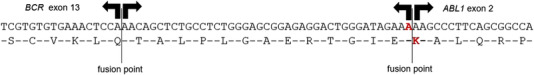
Fusion sequence of e13a1 on cDNA basis with the respective amino acid code. The deletion/insertion event resulted in an in‐frame fusion of the *BCR* and *ABL1* genes. In red bold letters, the G>A mutation in the *ABL1* exon 1 sequence insertion is shown resulting in an amino acid chance from glutamine to lysine (E27K).

In addition, the fusion of the 37 bp intron sequence (instead of the missing 38 bp *BCR* exon 13 fragment), to the last four bases of *ABL1* exon 1, resulted in the in‐frame fusion NM_004327.4:c.2670_2707delinsAACAGCTCTGCCTCTGGGAGCGGAGAGGACTGGGATAGAAA of *BCR* and *ABL1* (Figure 3). Because no frameshift occurred, it is assumed that the resulting fusion gene leads to functional BCR‐ABL1 protein synthesis and is capable of propagating CML. To what extent the genetic rearrangement has an influence on the patient's response to imatinib therapy is unclear since the patient ranged from MR4.5 to MMR during monitoring. Furthermore, a point mutation in the *ABL1* exon 1 sequence leads to the exchange of glutamic acid against lysine (E27K). The potential impact of this amino acid exchange on protein folding and activity requires further investigations. For future monitoring, the qRT‐PCR method for this patient has to be performed by LC instead of the TM PCR system.

## 4 CONCLUSION

Our scenario highlights the importance of an individual characterization of the *BCR‐ABL1* fusion sequence in case of unclear qRT‐PCR results. It is of high advantage if various validated detection methods are available in parallel in diagnostic laboratories so that the occurrence of *BCR‐ABL1* transcript variants and changed primer binding sites do not have a negative influence on therapy decisions.

## AUTHOR CONTRIBUTIONS

Naumann N contributed to manuscript drafting, data curation, and evaluation and reviewed the literature. Spiess B contributed to manuscript drafting, reviewed the literature, and was involved in supervision. Bross‐Bach U provided patient material and reviewed the manuscript. Seifarth W, Fabarius A, Hofmann W‐K, and Saußele S contributed to manuscript drafting, reviewing, and supervision. All authors issued final approval for the version to be submitted.

## FUNDING

For the publication fee, we acknowledge financial support by Deutsche Forschungsgemeinschaft within the funding program “Open Access Publikationskosten” directed by Heidelberg University.

## CONFLICT OF INTEREST

SS received honoraria from Novartis Pharma GmbH, Bristol‐Myers Squibb (BMS), Pfizer, ARIAD, and research funding from Novartis Pharma GmbH and BMS. All other authors declare no conflict of interest.

## ETHICS STATEMENT

The study design adhered to the tenets of the Declaration of Helsinki and was approved by the relevant institutional review board (Medical Faculty Mannheim, University of Heidelberg, 2013‐509N‐MA and 2020‐593N). The patient gave written informed consent.

## Data Availability

All data that support the findings of this study are included in the manuscript.
